# An intervention design for promoting quality of life among patients with multiple sclerosis: a protocol with a planning approach for a mixed methods study

**DOI:** 10.1186/s12883-023-03078-w

**Published:** 2023-01-26

**Authors:** Atefeh Homayuni, Zahra Hosseini

**Affiliations:** 1 Student Research Committee, Hormozgan University of Medical Sciences, Bandar Abbas, Iran; 2 Health Education and Health Promotion, Social Determinants in Health Promotion Research Center, Hormozgan Health Institute, Hormozgan University of Medical Sciences, Bandar Abbas, Iran

**Keywords:** Intervention, Multiple sclerosis, PRECEDE-PROCEED model, Quality of life

## Abstract

**Background:**

Multiple sclerosis is a chronic progressive disease of the central nervous system that affects the patients' quality of life. The disease's complications reduce the quality of life in patients by creating physical, psychological, social and economic problems for the patient and his/her family and reducing the patient's individual and social functioning. The aim of the present study is designing, implementing and evaluating an intervention based on the PRECEDE-PROCEED model to promote the quality of life in people with multiple sclerosis. This paper summarizes the study protocol.

**Methods:**

We will use the PRECEDE-PROCEED model for designing the study. In the first step, the factors affecting quality of life in people with multiple sclerosis will be determined by a qualitative study. In the second step, these factors will be prioritized based on their importance and variability, then behavioral and environmental factors of the most important priority will be identified. In the third step, the predisposing, enabling and reinforcing factors related to the identified priority will be determined by a qualitative directed content analysis. In the fourth step, a questionnaire will be designed and psychometric based on the results of the previous step. The fifth step will be about planning to implement the intervention. In the sixth step, the intervention will be implemented and its effectiveness will be evaluated by process, impact and outcome evaluations.

**Discussion:**

The results of this study will provide information about patients' needs and concerns and thus will contribute to policymakers, government, community, health professionals and families to take the necessary measures to improve quality of life in these patients.

## Background

Multiple sclerosis (MS) is a chronic inflammatory demyelinating disease of the central nervous system (CNS). Common symptoms of MS include: sensory symptoms (such as pins and needles, tingling or numbness), weakness, visual disturbances, diplopia, imbalance, gait disorders, dizziness, spasm, ataxia, nystagmus, neuropathic pain, urinary urgency or retention, sexual dysfunction, depression, emotional-cognitive disorders and inability to tolerate heat [1]. It is the most common non-traumatic disabling disease to affect young adults, with symptoms typically appearing in those aged between 20–40 years [2, 3]. Women are disproportionately affected by MS and are diagnosed two to three times more often than men [4]. The prevalence of MS varies worldwide and is highest in North America, Western Europe and Australia, and lowest in countries centered around the equator [5]. A total of 2.8 million people are estimated to live with MS worldwide (35.9 per 100.000 population) [6]. In Iran, a recent study found that Iran's Health Ministry recorded 36,287 confirmed cases of MS between 2010–2016, of which 8202 (22.6%) are male and 28,085 (77.4%) are female [7]. According to the latest statistics published by Iran MS Society in March 2022, the number of patients (who are members of Isfahan MS society) is 7476, including 5674 female patients and 1489 male ones. In this ranking, in terms of the disease prevalence, Isfahan is in the third position in the country [8].

Studies have shown that the manifestations of the disease can affect the quality of life (QOL) in these patients negatively, so that the complications caused by the disease impose mental, social, and economic pressures on the patient and his/her family addition to physical discomfort. This disease reduces the patients' personal and social performances, and as a result, has a significant impact on how they play their roles in life, their occupational status, and eventually their QOL (i.e. physical and mental health) [9]. The World Health Organization (WHO) defines QOL as "an individual's perception of their position in life in the context of the culture and value systems in which they live and in relation to their goals, expectations, standards and concerns" [10]. MS has created new demands and challenges, and people with MS experience unintended loss of roles, changes in relationships, and possible financial problems. These aspects of the disease have a direct impact on the patient’s psychosocial life, lead to a decrease in the QOL and problems in dealing with this situation and conditions [11]. Research findings showed that QOL in people with MS is lower than for healthy subjects and other chronic diseases [12].

Using a model as a framework for identifying the factors involved in maintaining/increasing the QOL in these patients, as well as designing interventions to promote their QOL is very important. The literature review has shown the efficiency of the PRECEDE-PROCEED model in predicting and promoting the QOL in different groups of people [13–18]. This model has been introduced by Green et al. as a diagnostic framework for health education and health promotion programs [19]. This model has 8 steps including: social assessment, epidemiological and behavioral assessment, educational and ecological assessment, administrative and policy assessment and intervention alignment, implementation, process evaluation, impact evaluation and outcome evaluation [20]. The model's comprehensive nature makes it possible to be employed in different subjects and populations. This model has an ecological viewpoint and states that it should not only be paid attention to the individual to change the behavior, but the environment around him/her and effective factors for behavior change should also be considered. In this model, in addition to the fact that a person should receive training to change his/her behavior, the supportive environments must also be considered for this behavior change [21].

According to the reviews performed by the researcher regarding the literature review in Iran and other parts of the world related to the QOL in people with MS, it was found that so far, it has not been done any study using the PRECEDE-PROCEED model. According to what was mentioned, the purpose of this study will be designing, implementing and evaluating an intervention based on the PRECEDE-PROCEED model to promote the QOL in people with MS.

## Methods

To design and direct the study, we will create a research team involving 3 experts in health education and health promotion, a neurologist, an expert in rehabilitation counseling, a clinical psychologist and a statistical specialist. In the first and third steps of the study, the planner will engage the patients in partnership to build the program and link the patients’ concerns about QOL issues to the program objectives. We will perform 2 focus groups involving 18 patients with the aim to determine the factors affecting the QOL in people with MS. After determining the factors affecting the QOL in the first stage, the final priority for the intervention will be determined based on a collaborative approach with the participation of patients, their caregivers and officials of MS Association. In the third step, we will perform individual interviews with patients with the aim to identify the factors associated with performing the identified behavior in the second step (i.e., predisposing, enabling and reinforcing factors). In the fifth step, the decision about how to conduct the intervention (time to start the intervention sessions, the best platform for holding sessions, the number of sessions, time duration of each session, duration of the intervention, suggested intervention strategies, methods of participation and cooperation of patients in sessions, …) will be reached after consultation with patients. In this step, we will invite participants to build educational materials (such as clips), do the educational assignments and participate in Q&A meetings.

**Fig. 1:**
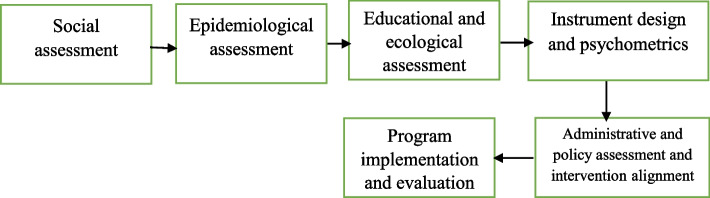
Stages of promoting QOL in people with MS

The main stages of study are presented in Fig. 1.

The approximate timeline of the project is presented in Table 1.

**Table 1:** Project's timeline

**Step**	**Activities**	**Duration (month)**
***Social assessment:*** Identifying the factors affecting the patients' quality of life	-Conducting 2 focus groups with the participation of patients, their caregivers and officials of MS Association	5 months
***Epidemiological assessment:*** Prioritizing the identified factors based on the importance level and variability	-Prioritizing the factors affecting the QOL in terms of importance and variability -Identifying the behavioral and environmental factors of the identified priority (the health problem) -Prioritizing each of these factors in terms of importance and variability	1 month
***Educational and ecological assessment:*** Identifying the predisposing, enabling and reinforcing factors	-Doing individual interviews with patients	5 months
***Instrument design and psychometrics***	-Designing the basic draft of the questionnaire based on the data gathered through individual interviews in the last step -Removing the duplicate items and merging and modifying the other items -Examining the face validity and content validity of the initial proposed questionnaire -Examining the construct validity (performing exploratory and confirmatory factor analysis) -Examining the reliability	3 months
***Administrative and policy assessment and intervention alignment***	-Conducting a pre-test survey -Conducting the educational needs assessment based on the information obtained from the pre-test results -Designing the educational intervention (determining the number of sessions, time duration of each session, resources, human resources, budget, educational materials, …)	1 month
***Program implementation***	-Conducting the intervention sessions based on the intervention plan designed in the previous step	2 months
***Waiting time to make behavioral changes***		6 months
***Evaluation***	-Conducting process evaluation (assessing the learners' progress and their temporary achievement in the program goals, assessing training and trainer qualities, training contents, …) -Conduction impact evaluation (assessing the predisposing, enabling and reinforcing factors) -Conducting outcome evaluation (conducting post-test survey 6 months after the intervention)	1 month

### Step 1: Social assessment

In this step, an assessment of patients' perception of their needs and QOL will be done. In fact, in this step, the planner's task is to perceive the social problems in the same way as the patients. Social assessment gives planners the possibility to assess the power and capacity of problem solving in the target group and design the interventions to be led the promotion and expansion of the group to solve the problem [22]. In this step, in order to identify the general, health, and social problems perceived by the study population (people with MS), their QOL will be evaluated. A qualitative study with a content analysis approach will be done to explore the patients' QOL accurately as possible and identify the factors affecting it. Then, the results of this study will be summarized and analyzed.

### Study design

A qualitative methodology will be used to identify participants’ experiences and perspectives about factors affecting their QOL. This study will be conducted with conventional content analysis approach.

### Study sample and recruitment

People with MS who will refer to Isfahan MS Association and their caregivers will be participated in this study. Participants will be selected using a purposive sampling and maximum diversity (in terms of age, gender, education, marital status, employment status, different types of the disease, including relapsing remitting, primary progressive and progressive, etc.). After obtaining the required permission from the head of research affairs of the MS Association, various methods will be used to enroll the participants. These methods include: posting an advertisement on the MS Association website, installing an advertisement in the MS Association and making direct personal interactions with patients. The researcher will explain about the purpose of the study, inclusion criteria and data collection method in advertisements. Individuals interested to participate in the study will be contacted by the researcher by telephone and the necessary arrangements will be made to interview those who may be willing to participate in the study. Data collection will be continued until data saturation. To ensure voluntary participation in the study, all participants will receive consent forms to sign. They will be assured that the transcript of the interview would remain strictly confidential and that they would not be named in the final description and analysis.

### Inclusion and exclusion criteria

In order to select the participants in different steps of the study, we will use some inclusion and exclusion criteria. These criteria are presented in Table 2.

**Table 2:** Study's inclusion and exclusion criteria

Step number	Inclusion criteria	Exclusion criteria
Steps 1 & 3	1) Having MS diagnosed by a neurologist; 2) Having MS for more than 1 year; 3) Having not a chronic disease other than MS; 4) Being able to participate in the interview and sharing their experiences; and 5) Having rich and useful experiences about living with the disease and willing to retell their experiences to the researcher (*only in step 1*)	Individuals will be excluded if: 1) Being unable to cooperate and talk due to the worsening of the disease or other reasons; 2) Not willing to continue the interview at the time of the interview; and 3) Having cognitive dysfunction or severe disability
Steps 4 & 6	1)Having MS diagnosed by a neurologist; 2) Having MS for more than 1 year; 3) Having not a chronic disease other than MS; 4) Willingness to participate in the study; 5) Internet access to answer questions; and 6) Access to the virtual networks of WhatsApp or Telegram and the ability to use it (*only in step 6*)	1) Having cognitive dysfunction or severe disability; and 2) If they lost any of the inclusion criteria

In the first step, since it seems that some participants will not be able to be interviewed (due to having speech problems and other reasons), so the researcher will interview with their family caregivers. Some of the criteria for selecting the family caregivers will include the followings: individuals who will 1) be member of a family as a sibling, parent, spouse or child of patients for more than 1 year and 2) be responsible for taking care of an individual with MS.

### Data collection

Each participant will take part in a semi-structured interview which is estimated to last between 30 to 90 min. Interviews will be conducted in a quiet room in the MS Association or participants’ home according to the preferences or convenience of the participants. An initial interview guide was developed for this study. The interview guide will include two parts: the first part will ask questions about participants' demographic information (including age, age of disease onset, education level, marital status, and so on). The second part will include semi-structured open-ended questions. Each interview will be started by asking these main questions in the interview guide: 1) How has this disease affected your life? 2) What are the most important factors that you think affect the quality of your life? 3) What are some of the factors that have contributed to improve your QOL? 4) What factors are undermining your QOL? 5) What are you currently doing to improve your QOL? 6) What do you believe are the facilitators to QOL for people with MS in the community in the individual, organizational and policy levels? (Caregivers version) [23]. In-depth and exploratory questions will be asked to elaborate on the details.

### Data analysis

Data collection and analysis will be carried out simultaneously. Interviews will be audio-typed with participants' permission, will be handwritten on the paper and then will be typed as soon as possible. Data will be analyzed using MAXQDA-10 software. Each transcript of interview will be read several times to identify the concepts hidden in the statements of the participants. After extracting the initial codes, codes will be clustered into categories, based on their similarities. The process of analysis will be continued until the emergence of the main and sub-categories.

### Rigor

To ensure the trustworthiness of findings, the researcher will use the criteria suggested by Lincoln and Guba [24]: 1) reviewing the extracted manuscripts and codes by participants, 2) sending the data to colleagues, using their supplementary comments and revising the findings based on their comments, 3) continuous reviewing and devoting sufficient time for data collection, 4) selecting participants with maximum diversity, 5) describing the research processes and participants' characteristics in detail.

### Step 2: Epidemiological assessment

Epidemiological assessment addresses which health problem is of significant importance for the patients [22]. This step aims to prioritize the factors affecting the ones' QOL based on the importance level and variability, identify the behavioral and environmental factors of the identified priority, and prioritize each of these factors in terms of importance and variability. After identification and extraction of factors affecting the patients' QOL, based on the participants' experience, the list of patients' needs will be gathered. Based on the review of texts, science resources, and theses/ dissertations, these factors and needs will be prioritized based on the importance level and variability, and the most important factors will be identified. In the next stage, after the health problem is accurately identified, the list of behavioral and non-behavioral factors in such a way that involved in the creation of this priority will be provided. Non-behavioral factors are the personal and environmental factors which exist in the community but are not related to the one's behavior and affects the health indirectly (such as environmental, economic, social, genetic, … factors). Conversely, behavioral factors are those that affect the health and well-being of the community. In this step 5 fundamental stages will be conducted as following:

1)Discriminating between behavioral and non-behavioral causes of the health problem; 2) Providing the list of behaviors: in this stage, two groups of behaviors are determined. The behaviors and functions which prevent creating the health problem, and behaviors and treatment methods; 3) Ranking the behaviors in terms of the importance level; 4) Ranking the behaviors in terms of variability; and 5) Choosing the target behavior: to adjust the behaviors based on the importance and variability, the behavior on which educational interventions will focus, will be identified and selected [25]. In the following, according to the existing facilities, time duration of the project, and participatory prioritization, final prioritization for the intervention will be determined. In the classification of behaviors in terms of importance and variability, the behaviors should be considered that are related to the health problem apparently and educational programs succeeded in changing them.

### Step 3: Educational and ecological assessment

The third step guides the planner towards determination of primary and reinforcing factors. These factors must be existed due to the process change that is begun and continued. These factors have been classified as predisposing, enabling, and reinforcing factors, and overall affects the possibility of occurring environmental and behavioral changes. Division of factors affecting the performance of the health behavior to predisposing, enabling, and reinforcing factors will cause the subject's needs and wants to be considered [22]. In this section of the study, we will examine and analyze patients' opinions about the factors associated with performing the identified behavior in the prior step (predisposing factors, enabling factors and reinforcing factors).

### Study design

This study will be based on a qualitative directed content analysis. In this approach, the initial coding starts from a theory, and the selected theory can help to focus on the research question [26].

### Study sample and recruitment

Based on purposive sampling with maximum diversity (similar to the first step), patients will be selected among those who will refer to the Isfahan MS Association.

### Data collection

The data collection process in this step is the same as the first step and will continue until the data saturation. At the beginning of each session, the interviewer will introduce herself and explain about the interview and the objectives of the research. The interviews will be recorded by a voice recorder. The interview process will begin with questions about the participants’ demographic information. The interview questions will be based on the constructs of the educational factors of PRECEDE model, which will begin and continue with the following questions: 1) Tell me about your skills and abilities to do X behavior? (X refers to the behavior that is identified in the previous step and in this step we want to identify the reinforcing, enabling and predisposing factors for this behavior). 2) In your opinion, what skills should you learn to do X behavior? 3) Tell me about your experiences regarding environmental barriers for doing the X behavior. 4) Tell me about your experiences regarding the role of family members, health care providers, friends, and others in performing this behavior. 5) Tell me about your feelings after doing this behavior continuously. 6) What problems did you have, when performing this behavior?

### Data analysis

Interviews will be first handwritten on the paper and then will be typed as soon as possible. Since the purpose of the research is to identify and categorize all cases related to a particular phenomenon, the entire text will be read and those sections will be marked that are specified based on the researcher’s initial impression. In the next stage, the marked sections will be coded based on predetermined codes (according to the theory). A new code will be given to each section of the text that does not fit into this initial coding. To facilitate the organization and analysis of the qualitative data, the MAXQDA version 10 software will be used.

### Step 4: Instrument design and psychometrics

In this part of the study, after extracting the concepts, primary codes and classes, primary themes and items will be identified and the proposed instrument will be formulated. The items will be addressed and reviewed to ensure that they do not have overlap and duplication. Then, the repetitive items will be removed and the ones which can be integrated, merged. Next, validity and reliability of the proposed instrument will be measured. To determine validity of the instrument, 3 methods of face validity (two qualitative and quantitative methods), content validity (two qualitative and quantitative methods), and construct validity will be used. To address reliability of the instrument, internal consistency (Cronbach's alpha coefficient) and stability reliability (test–retest) methods will also be used.

### Study sample and recruitment

In this part of the study, for psychometrics and measurement of validity of the tool, a panel of experts with the number of 10 people of different specialties (including psychology and counseling, neurology, health education and promotion and qualitative research experts) will be used. After the analysis of views and design of the proposed primary instrument, the designed questionnaire for examining the construct validity and face validity will be given to the people with MS to be completed.

The minimum number of samples required for confirmatory factor analysis is 5 people for each question [27]. The study population will include all people with MS who will refer to the MS Association and Charity Foundations for Special Diseases in Isfahan.

### Data collection

We will use online questionnaires to collect data. The questionnaire will be designed virtually and the link of the questionnaire will be placed on the Telegram and WhatsApp channels of the MS Association and Charitable Associations.

### Data analysis

Data will be analyzed using SPSS-24 and EQS6.1 software.

### Step 5: Administrative and policy assessment and intervention alignment

In this step, the educational strategies affecting the predisposing, enabling, and reinforcing factors will be selected. In other words, we will conduct a pre-test survey with the questionnaire that was designed in the previous step. Based on information obtained from this, educational needs assessment will be conducted and then an educational intervention will be designed in order to train patients. This step aims to identify the essential policies to launch a program, existing and required resources, as well as possible obstacles affecting the program performance. The intervention will be designed based on information obtained from pre-test stage and constructs of the predisposing, enabling and reinforcing factors.

Here the number of meetings, time duration of each meeting, duration of the intervention, resources, human resources, and budget will be determined by the research team according to needs of the testing group. Finally, all obstacles will be reviewed and suitable solutions given. Also, any organizational policy, regulations, and laws which may affect the program performance, must be considered and a solution given to it.

Due to the prevalence of coronavirus in the world and in order to protect the health of participants, it is predicted the intervention meeting is held in an online form via virtual networks of Telegram and WhatsApp. Some interventions that their performance is predicted, it can be referred to the cases as follows:

1. Using short voices, images, films, and clips related to the desired behavior. All of these teaching materials will be prepared by the research team.
2. Presenting the educational assignments at the end of each educational meeting and practices to the instructor to receive a feedback;
3. Holding question-answering meetings online;
4. Designing and presenting a poster and an educational booklet;
5. Introducing the educational help books if necessary;
6. Attending at least one of the patients' family members in the educational meetings, etc.

### Step 6: Program implementation and evaluation

In the end, after the program implementation, there will be a need to determine efficiency of the intervention program. In this way, a quasi-experimental study will be designed and implemented. The study population will be people with MS who will refer to MS Association and Charity Foundations for Special Diseases in Isfahan.

### Sample size

Based on the previous studies, the standard deviation of the score of the quality of life is 11.01. Considering the error of 5%, the testing strength of 80%, and difference of 5 for the score of two intervention and control groups, the sample size was calculated in each group as follows:

$$n = \frac{{2*(z_{1 - \alpha /2} + z_{1 - \beta } )^{2} \sigma^{2} }}{{d^{2} }} = \frac{{2*(1.96 + 0.84)^{2} *(11.01)^{2} }}{{(5)^{2} }} \approx 76$$

To prevent possible fall, we add 10% to the size of the above sample. Therefore, the size of the final sample was estimated 83 people in each group and 166 people in total.

### Participants recruitment

The data collection process in this step will be similar to step 4. Before collecting the information, the necessary descriptions regarding the research goals and how the participants participate in this research, will be presented for the people and ones tend to participate in, will answer to these questions. In this step, data will be gathered once before beginning the educational intervention and once 6 months after ending the educational intervention in both control and intervention groups.

### Instrument

The tools used in this part include the demographic questionnaire and the final version of the questionnaire that will be designed, validated, and measured in the fourth step [28]. On the one hand, it is expected that the patients' QOL is changed by providing interventions. So, the questionnaire of Multiple Sclerosis Quality of Life (MSQoL-54) will also be used.

- Demographic characteristics questionnaire: In this section, demographic information will be measured by variables such as: gender, age, age at the onset of disease, marital status, education level, occupation status, the disease history in the relationships, type of the disease (the disease course) and access to the treatment (type and the length of treatment).
- MS Quality of Life Questionnaire (MSQoL-54): This questionnaire has been made by Vickrey in 1995 [29]. In this questionnaire, different aspects of the QOL in people with MS (including: physical health, role limitations due to physical problems, role limitations due to emotional problems, pain, emotional well-being, energy, health perceptions, social function, cognitive function, health distress, sexual function, change in health, satisfaction with sexual function and overall QOL) are assessed. Finally, the score of the patient's QOL will be determined by scores of two combined aspects including "physical health composite" and "mental health composite". Scores of all 14 aspects as well as 2 ones combined ranges from 0 to 100. The higher scores imply the better situation. Scoring this questionnaire is performed in a Likert scale from 2 to 7 [30]. In the Persian version of this questionnaire, Cronbach's alpha has been estimated 0.926. The scale has convergent validity in each aspect successfully. Eventually, construct validity of the questionnaire has been confirmed using the factor analysis [31].

### Statistical analysis

Data will be analyzed using SPSS 19 software. Independent and dependent t-tests and analysis of covariance will be used to analyze the data. The p < 0.05 will be considered as statistically significant.

### Evaluation

The evaluation of the educational program will be done in 3 levels of the process evaluation, outcome evaluation, and impact evaluation. In the process evaluation, the process of doing different stages of education will be compared with the existing standards. In this kind of the evaluation, the level of learners' progress and their temporary achievement in the program goals will be assessed through the homework assignments. Also, it will be tried to obtain an assessment of training time, how the training is hold, training and trainer qualities, training content, the strengths and weaknesses of the program and impact of education with a survey of participants. This evaluation allows the educational program to be consistent with the learners' educational needs and coordinated with the predefined targets. In this study, the impact evaluation will be reviewed by studying the situation of predisposing, enabling, and strengthening factors via a designed and psychometric questionnaire in the fourth stage of the research. And finally, we will study how much the patients' QOL has improved via the participants' statements and result analysis of the QOL questionnaire which will be completed by them spontaneously before and 6 months after the educational intervention.

## Discussion

The present study will be conducted with the aim of designing, implementing and evaluating an intervention based on the PRECEDE-PROCEED model to promote QOL in people with MS in Isfahan. The literature review in Iran and out of this country shows that so far, no study has investigated the patients' QOL with MS using the PRECEDE-PROCEED model, and the studies that has examined the subject in other diseases and fields such as type 2 diabetes [17], elderly [18] and rheumatoid arthritis [13] using this model are limited to cross-sectional or intervention studies. Therefore, based on the patients' experience with MS, the results of this research will help policymakers, managers in charge of planning at the level of the Ministry of Health, managers of charity societies, doctors, medical staff, etc. to formulate effective plans and interventions to promote the patients' QOL.

Ethical approval was received for this study from the Ethics Committee of the Hormozgan University of Medical Sciences (IR.HUMS.REC.1399.065). A written consent will be obtained from the participants for participating in this study. They will be assured about the confidentiality of their information.

### Limitations of the study

Our study has also potential limitations that we need to consider. One of the limitations of this study is the use of self-report measures. Nondisclosure and social desirability concerns may affect reporting accuracy. To reduce the chance of this occurring, participants will be assured that their information will remain confidential with the researcher. A further limitation of this study is selecting patients who will have access to internet and are able to respond online. Thus, we may lose patients who do not have access and those who have motor and mental limitations. To resolve this limitation, we will identify these patients, who are interested in participating in the study through an online advertisement posted on the MS Association website, installing a printed advertisement in the MS Association and direct personal interactions with patients in the MS Association. By creating a suitable environment, we will try to hold the training for these patients in person and they will be able to respond to the questionnaires with the help of a member of their family or association employee. One of the positive points of this research will be the use of the experiences of patients who are members of the MS Association along with the experiences of patients who do not benefit from the services of the MS Association.

## Data Availability

Not applicable.

## Abbreviations

CNS: Central nervous system
MS: Multiple sclerosis
MSQoL: Multiple sclerosis quality of life questionnaire
QOL: Quality of life
WHO: World health organization
