# Correction: An intervention design for promoting quality of life among patients with multiple sclerosis: a protocol with a planning approach for a mixed methods study

**DOI:** 10.1186/s12883-023-03103-y

**Published:** 2023-02-06

**Authors:** Atefeh Homayuni, Zahra Hosseini

**Affiliations:** 1 Student Research Committee, Hormozgan University of Medical Sciences, Bandar Abbas, Iran; 2 Health Education and Health Promotion, Social Determinants in Health Promotion Research Center, Hormozgan Health Institute, Hormozgan University of Medical Sciences, Bandar Abbas, Iran

**Correction: BMC Neurol 23, 42 (2023)**

**https://doi.org/10.1186/s12883-023-03078-w**

Following publication of the original article [1], the authors reported an error in the author group. The correct author group are as follows: Atefeh Homayuni and Zahra Hosseini.

The author group has been updated above and the original article [1] has been corrected.
